# Mechanical Characterization of Liposomes and Extracellular Vesicles, a Protocol

**DOI:** 10.3389/fmolb.2020.00139

**Published:** 2020-07-21

**Authors:** Daan Vorselen, Melissa C. Piontek, Wouter H. Roos, Gijs J. L. Wuite

**Affiliations:** ^1^Fysica Van Levende Systemen and LaserLab, Vrije Universiteit Amsterdam, Amsterdam, Netherlands; ^2^Moleculaire Biofysica, Zernike Instituut, Rijksuniversiteit Groningen, Groningen, Netherlands

**Keywords:** (small) vesicles, liposomes, extracellular vesicles, atomic force microscopy (AFM), nanoindentation, bending modulus, Canham-Helfrich theory, mechanical properties

## Abstract

Both natural as well as artificial vesicles are of tremendous interest in biology and nanomedicine. Small vesicles (<200 nm) perform essential functions in cell biology and artificial vesicles (liposomes) are used as drug delivery vehicles. Atomic Force Microscopy (AFM) is a powerful technique to study the structural properties of these vesicles. AFM is a well-established technique for imaging at nanometer resolution and for mechanical measurements under physiological conditions. Here, we describe the procedure of AFM imaging and force spectroscopy on small vesicles. We discuss how to image vesicles with minimal structural disturbance, and how to analyze the data for accurate size and shape measurements. In addition, we describe the procedure for performing nanoindentations on vesicles and the subsequent data analysis including mechanical models used for data interpretation.

## Introduction

Atomic force microscope (AFM) imaging and nanoindentation are well-established techniques for nanometer resolution imaging and the investigation of mechanical properties of a variety of different materials, ranging from cement-based materials (Hu and Li, 2015) and crystalline metals (Voyiadjis and Yaghoobi, 2017) to biological matter (Krieg et al., 2019). AFM approach has gained much attention, especially in the field of mechanobiology as it can be operated in close-to physiological environments and allows for quantification of the response of biological systems to physical forces (Engel and Gaub, 2008; Roos et al., 2010; Dufrêne et al., 2013; Piontek and Roos, 2018). Importantly, the mechanical properties extracted from nanoindentation can be directly linked to function. Several different biological systems have been characterized mechanically, e.g., at the single protein level as proteins and ligand-receptor bonds (Puchner and Gaub, 2009; Lo Giudice et al., 2019), at the macromolecular complex level such as viruses and protein shells (Roos et al., 2010; de Pablo and Mateu, 2013; Buzón et al., 2020) and at the cellular level, such as characterization of cancer cells (Lekka, 2016; Rianna et al., 2020). For a variety of other biological systems the mechanical characteristics have remained elusive. However, this is quickly changing and for instance the mechanical properties of nanoscale vesicles are now increasingly being unveiled.

Vesicles are membrane-enclosed compartments that are abundant in biology and are found both inside and outside of cells. Extracellular vesicles are released by prokaryotic and eukaryotic cells and are present in all kinds of body fluids, e.g., blood/plasma (Chargaff and West, 1946), human saliva, breast milk (Lässer et al., 2011) and urine (Pisitkun et al., 2004). Small vesicles (<200 nm) are also involved in several important functions in cell biology, such as intracellular trafficking and membrane protein recycling (Maxfield and McGraw, 2004), transmission of signals in the neural system by synaptic vesicles (Südhof, 2004) and intercellular communication by extracellular vesicles (Camussi et al., 2010). The latter are suggested to play a role in cancer progression and could serve as an early biomarker for cancer (Melo et al., 2014; Costa-Silva et al., 2015). Other pathologies in which vesicles have been reported to be involved, are diabetes and multiple sclerosis (György et al., 2011). Furthermore, many viruses, such as influenza, HIV and Ebola, are surrounded by lipid envelopes. In drug delivery, vesicles in this size range are being intensively studied as drug delivery vehicles (Vader et al., 2016) and have already been clinically approved as synthetic nanocarriers for drugs (Allen and Cullis, 2013).

Membranes, including small vesicles, are subjected to mechanical stresses that lead to changes in shape during their lifetime. For example, exocytosis, endocytosis and fusion, and transport are all processes in which membranes are deformed. Theoretical models (Yi et al., 2011) and molecular dynamics simulations (Yue and Zhang, 2013) suggest that the nanoparticle's stiffness could affect endocytosis. Concomitantly, experimental studies also showed that particle stiffness can alter endocytic pathways (Banquy et al., 2009), efficiency of uptake (Kol et al., 2007; Anselmo et al., 2015) and circulation time in the blood (Anselmo et al., 2015). Thus, the mechanical characterization of vesicles is of outmost interest for elucidating their physiological and pathophysiological role and functioning. It has been shown that lipid composition and membrane proteins can change the stiffness (Rawicz et al., 2000; Dimova, 2014; Sorkin et al., 2018) and intrinsic radius of curvature of a membrane (McMahon and Gallop, 2005; Graham and Kozlov, 2010; McMahon and Boucrot, 2015). Indeed, it has been found that small vesicles are often enriched in specific lipids and proteins, such as the HIV virus envelope (Aloia et al., 1993) and exosomes (Théry et al., 2009; van Dommelen et al., 2012).

Although there are multiple well-established methods for extraction of mechanical information of micrometer-sized vesicles, e.g., fluctuation spectroscopy, micropipette aspiration and membrane tether pulling, AFM is currently the only method allowing for the mechanical characterization of submicrometer-sized vesicle populations (Piontek et al., 2019). It provides high resolution images of small vesicles on a single particle level under close-to physiological conditions and allows for performing force spectroscopy analysis. AFM imaging of vesicles has been employed to characterize the size and shape of individual natural vesicles (Laney et al., 1997; Sharma et al., 2011; Regev-Rudzki et al., 2013; Melo et al., 2014), interaction with surfaces (Bakowsky et al., 2008), rigidity of vesicles (Nakano et al., 2008) and to understand formation of supported lipid bilayers from liposomes (Reviakine and Brisson, 2000; Richter and Brisson, 2005). Next to imaging of vesicles, nanoindentations have been used to reveal mechanical properties of single vesicles (Laney et al., 1997; Liang et al., 2004b; Li et al., 2011; Calò et al., 2014; Vorselen et al., 2017; Benne et al., 2020).

Although the research field of small vesicles has grown enormously during the last decades, and several attempts have been made to extract their mechanics, the understanding of the mechanical properties of small vesicles remains limited (Piontek et al., 2019). This is among others due to the non-standardized experimental procedures and data analysis, including application of different models, e.g., Hertz, Sneddon or thin shell models (Krieg et al., 2019). Here, we present a standardized AFM procedure specifically for imaging and indenting single small vesicles under physiological salt conditions, including surface preparation and data analysis based on Canham-Helfrich theory (Vorselen et al., 2017). This model accounts for the internal pressure built up upon substrate adhesion, which is often neglected. This approach has already shed light on the influence of the lamellarity and size on the mechanical properties of small vesicles (Vorselen et al., 2017, 2018a), on the role of membrane proteins in mechanics of liposomes and extracellular vesicles (Sorkin et al., 2018, 2020), and revealed mechanical dependence of erythrocyte extracellular vesicles on pathological state (Vorselen et al., 2018b). Although these procedures are specifically described for imaging and indentation of vesicles, several aspects of this approach are also beneficial for AFM-based study of other nanoparticles (e.g., block-copolymer vesicles, viruses and nanocages).

## Materials and Equipment

### Materials

- Mixture of 3 ml concentrated hydrochloric acid (HCl, 37 %) and 97 ml ethanol (96.2 %); to be scaled for the volume needed
- Poly-L-lysine solution (1 mg/100 ml), dissolved in Milli-Q water or demineralized water
- Milli-Q water for washing
- Glass coverslips (appropriate for the AFM system used)
- Teflon or glass rack to hold the coverslips separately
- Staining glass (+ lid) for the rack with coverslips
- Tweezers
- Pipettes
- Falcon tubes and Eppendorf tubes
- Oven or incubator (heated at 37°C)
- Cantilevers (e.g., qpBioAC (https://www.nanoandmore.com/AFM-Probe-qp-BioAC); please see section *Cantilever and tip selection* for further guidelines on tip selection)
- Phosphate-buffered saline (PBS).

### Equipment

- An AFM capable of force spectroscopy, including computer to control the instrument and an optical microscope/camera to align the laser and cantilever (note: a closed loop AFM system facilitates experiments with respect to time for imaging).

### Software

- AFM data analysis software (e.g., software provided by AFM manufacturer; alternatively Gwyddion [download: http://gwyddion.net/download.php]) or home-built scripts (e.g., in MATLAB or Python), or software for manual analysis (e.g., Origin), to analyze AFM images and force-distance curves.

### Methods

#### Vesicle Adhesion to the Surface

**Figure 1:**
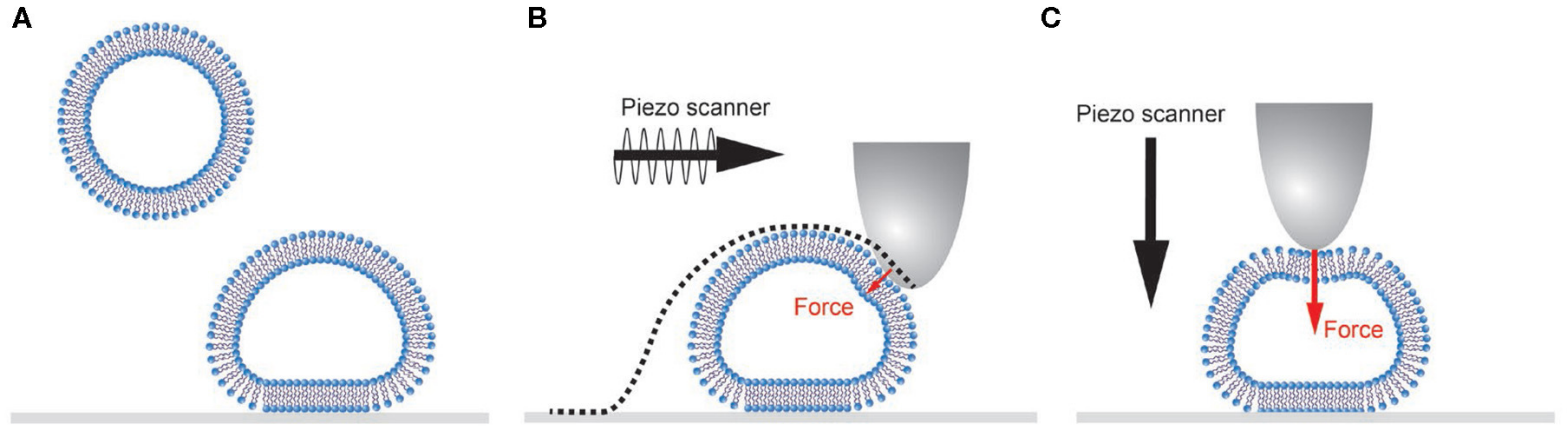
Schematic representation of single vesicle AFM experiments. **(A)** Vesicles adhere and subsequently deform onto a surface. **(B)** During AFM imaging the tip is scanned over the sample. In force distance-based imaging the cantilever is oscillated below its resonance frequency, and the force exerted on the sample is well controlled. Small forces will still result in non-negligible sample deformation. The recorded AFM image is always a convolution of the vesicle and tip shape (dotted line). **(C)** During a nanoindentation experiment the cantilever is lowered onto the center of the vesicle applying a higher force set point (typically 0.5–10 nN) than during imaging.

Vesicles are typically adhered to a surface based on non-specific interactions (Figure 1A). Natural vesicles often contain negatively charged lipids such as phosphatidylserine, so a positively charged surface (e.g., poly-l-lysine coated glass slides) results in binding based on electrostatic interaction. Because of the relatively small bending modulus of lipid bilayer membranes (10–50 *k*_b_*T*; Olbrich et al., 2000; Gracià et al., 2010), vesicles deform upon binding. The final shape that vesicles adopt is determined by a balance of the vesicle-surface adhesion energy, the bending and stretching energy of the membrane and the buildup of an osmotic pressure difference due to volume loss of the vesicle (Seifert and Lipowsky, 1990). Under equal or outward osmotic pressure, a spherical cap is the expected shape with minimized free energy.

Preparation of coated glass coverslips with poly-L-lysine

1. Prepare the poly-L-lysine solution (1 mg/100 ml). Leave it on a stirrer to dissolve the poly-L-lysine completely (5–10 min).
2. Place new glass coverslips in a Teflon rack in a staining glass.
3. Add the ethanol-HCl solution to the staining glass to cover the coverslips. Allow to stand for 10 min at room temperature. (During this step work in the fume hood).
4. Wash the slides two times with Milli-Q water, discarding the washes.
5. Place the slides into the poly-L-lysine solution and allow to stand for 1 h at room temperature.
6. Wash the slides briefly (1 or 2 s) in H_2_O (Milli-Q water) and dry them at least 5 h or overnight at 37°C.
7. The coated slides can be stored at 4°C for a maximum of 1 month.
8. After exposure to vesicles, glass coverslips should be discarded, and a new poly-L-lysine coated glass slide should be used.

#### Force Distance Curve-Based Imaging of Vesicles

**Figure 2:**
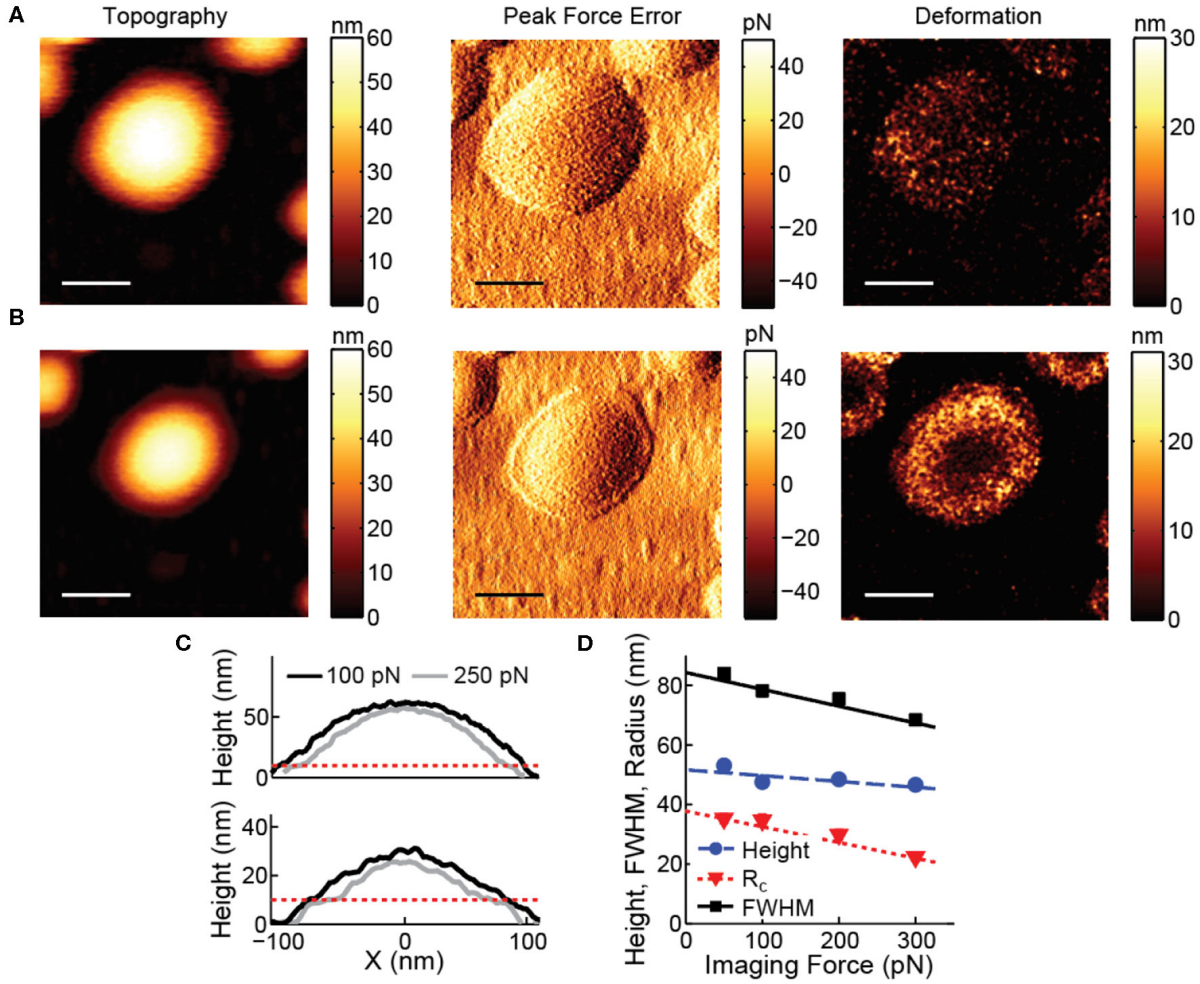
Effect of imaging force on vesicle images. **(A)** Topography, peak force error and deformation image recorded at 100 pN. **(B)** Topography, peak force error and deformation image recorded at 250 pN. All scale bars are 100 nm. **(C)** Comparison of line profiles through the maximum of the vesicles recorded at 100 and 250 pN. The dashed red line indicates the approximate height expected for a double lipid bilayer (~10 nm). Upper panel corresponds to the data in **(A,B)**. **(D)** Height, radius of curvature and full width at half maximum (FWHM) of liposomes recorded at various imaging forces. At least 180 particles were analyzed for each condition; error bars indicating standard error of the mean fall within the marker size. Lines show linear fits with slopes:−0.020 (Height), −0.056 (FWHM) and −0.053 (*R*_c_).

AFM imaging of vesicles is typically performed in tapping mode, to avoid disruptive high lateral forces. However, control of the forces normal to the surface in tapping mode is limited (Xu et al., 2008; Guzman et al., 2013). Peak forces often exceed 0.5 nN and result in considerable deformation (tens of nm) on top of the soft vesicles, potentially even leading to damage of the vesicles. The deformation is even larger on the sides of the vesicle. Correction for deformation is notoriously difficult, because it is influenced by exertion of higher normal forces on the side of the vesicle, the radial direction of the applied force and unknown response to force application on the side of the vesicle. Therefore, forces exerted by the AFM tip should be minimized. Exerted forces can be limited (<100 pN) using force distance curve-based AFM (Heinz and Hoh, 1999; Ortega-Esteban et al., 2012; Dufrêne et al., 2013; Pfreundschuh et al., 2014), allowing accurate measurement of size and shape of vesicles from images (Figure 2). In force distance curve-based AFM imaging the tip is oscillated below its resonance frequency, and the feedback is the deflection of the cantilever, essentially taking force distance curves at each pixel (Figure 1B). This results in constant and well-controlled peak forces exerted on the sample. To exemplify the negative effects of high imaging forces, Figure 2 shows the influence of a slight increase in imaging force and the resulting deformation on top of the soft vesicles and on the sides of the vesicle.

#### Nanoindentation of Vesicles

**Figure 3:**
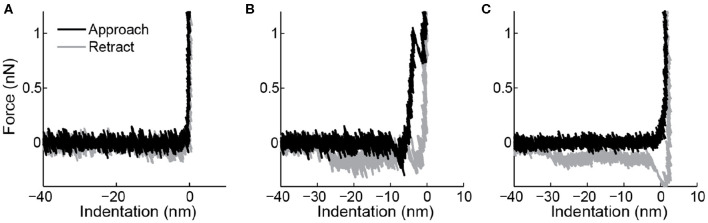
Force indentation curves taken on the surface. **(A)** Overlap between trace (black) and retrace (gray) and a sharp transition when the tip touches the surface suggest this is a clean tip. **(B)** A breakthrough event in the trace and a force plateau in the retrace indicating a lipid bilayer tether show that this tip was contaminated with a lipid bilayer. **(C)** This tip shows a non-abrupt change in slope when it hits the surface and again a force plateau in the retrace, indicating contamination with a lipid bilayer.

Nanoindentation is an established technique to get quantitative information about the mechanical properties of nanoparticles (Rosmalen et al., 2015; Marchetti et al., 2016). Here, we use a similar approach for vesicle indentations (see Figure 1C for a schematic representation). During such an experiment, first an image of the vesicle is made to characterize the geometry of the vesicle and the location of its center (maximum imaging force <100 pN). Before the vesicle is indented, a force distance curve on the substrate (force set point: 5–10 nN) next to the vesicle is recorded to demonstrate a clean tip and a linear response of the cantilever (Figure 3). Next, the AFM tip is moved to the center of the vesicle. First, a maximum force of 400–500 pN is applied to demonstrate fully elastic behavior of the vesicle. Subsequently, a higher force (5–10 nN) is exerted to the same location at least once. In all cases the force distance curves are recorded. After indenting the vesicle, another force distance curve pushing on the substrate next to the particle is recorded to check if the AFM-tip is still clean. Finally, another image is made to check for movement or changes in appearance of the vesicle (preferably with the same settings as the image before indentation). The nanoindentation is performed at a slow speed (typically 0.2–1 Hz), which results in a mostly elastic response and much better signal-to-noise ratio than the FZCs recorded during imaging. Repeated small indentations (up to ~0.5 nN) on the same vesicle typically yield quantitatively very reproducible behavior. Deeper indentation may cause vesicle damage, such as membrane rupture, which could lead to a different response upon repetition of indentation. Indentations of different vesicles are much less similar, as the indentation behavior depends on vesicle size and degree of spreading, which are likely to differ on a vesicle-to-vesicle basis.

It is known that lipid bilayers can adhere to AFM tips (Richter and Brisson, 2003), and under some ionic conditions even can form stacks on the tip (Pera et al., 2004). This results in changed surface chemistry and increased size of the indenter. The latter can have a large impact on indentation response of vesicles (Vorselen et al., 2017). Checking tip-cleanliness on the sample surface can prevent performing measurements with a contaminated tip. A force indentation curve of a clean tip is shown in Figure 3A. Typical marks for lipid bilayers adhering to the tip are bilayer penetration events (Figure 3B), a non-sharp transition when touching the surface (Figure 3C), or pulling of a lipid bilayer tether during retraction (Figures 3B,C). The latter is marked by a force plateau of 0.005–0.15 nN (Bo and Waugh, 1989; Armond et al., 2011).

To derive quantitative mechanical parameters, elastic models are fitted to the indentation response of vesicles. It is therefore important to demonstrate that the observed response is fully elastic. This can be achieved by making small indentations to confirm overlap between the approach and retraction curve (Vorselen et al., 2017). If there is hysteresis between approach and retraction there is a viscous component in the response, and the speed of the indentation should be lowered.

Step-by-step vesicle nanoindentation procedure

**Figure 4:**
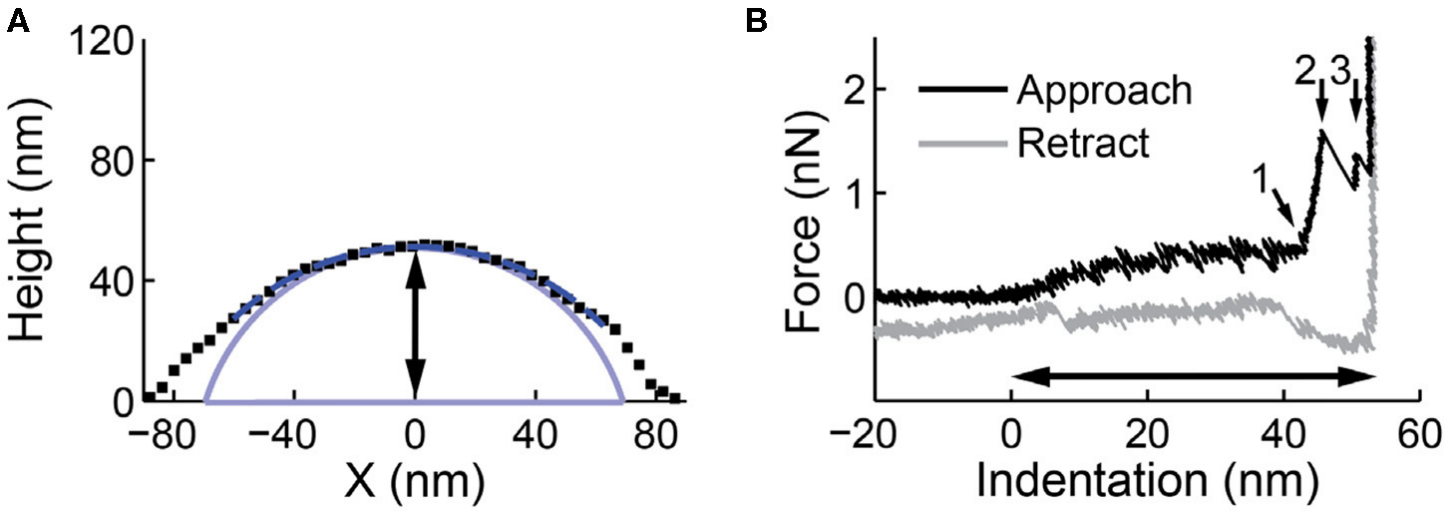
Line profile and nanoindentation on a liposome. **(A)** A line profile along the slow scanning axes through the maximum of the particle. A circular arc is fitted to the upper half of the line profile (dashed dark blue). The spherical cap shape after deconvolution is shown in light blue. The black arrow indicates the height derived from the image (51.9 nm). **(B)** A force indentation curve on the same particle. The contact point between the tip and the vesicle is set at 0 nm indentation. At around 45 nm, marked by small arrow 1, the two bilayers are pressed together. After two discontinuities (small arrow 2 and 3), the glass surface, which appears infinitely stiff, is reached. The black arrow indicates the height derived from the indentation curve (55.6 nm), which is a more accurate (and typically larger) estimate than the height estimation from the profile in **(A)**.

Note: The nanoindentation is performed at a slow speed (typically 0.2–1 Hz), and kept constant for all indentations. Additionally, all force distance curves recorded consist of an approach and retract curve.

1. Record an image of a single vesicle (maximum imaging force <100 pN).
2. Record a force distance curve (FZC) on the substrate (force set point: ~5 nN, or higher if needed for bilayer penetration, which depends on tip size and membrane composition) next to the vesicle.
3. Indent the center of the vesicle and record the corresponding FZCs:
  - Initially with a maximum force of 400–500 pN.
  - Next, apply a higher force (5–10 nN) to the same location at least once. Make sure to penetrate both lipid bilayers and reach the underlying substrate in the high force indentations, which allows accurate measurement of the vesicle height (Figure 4). Also ensure to retract the cantilever tip well beyond the vesicle contact point (~250 nm) to allow a lipid tether to form and break during the retraction, which is critical for full mechanical analysis of the vesicle response (see section *Analysis of Force Indentation Curves*).
4. Record another force distance curve pushing on the substrate (~5 nN).
5. Make another image of the vesicle (preferably with the same settings as those used for the image before indentation).

#### Image Analysis for Accurate Size and Shape Measurement

The recorded image in AFM is always a convolution between the sample and the tip (Figure 1B). During the indentation of vesicles, the usage of very sharp tips (*R*_t_ <5 nm) may cause the integrity of the vesicle to be compromised, hence larger tips (*R*_t_ = 10–15 nm) are advised. Assuming a spherical membrane cap shape (Seifert and Lipowsky, 1990), the vesicle shape can be deconvoluted using simple geometric arguments. This is especially important for vesicles that do not deform much onto the surface, where the tip-broadening artifact is largest. The radius of curvature *R*_c-_ can be obtained by fitting a circular arc ($-R_{\text{c}}-R_{\text{t}}+H+\sqrt{{\left(R_{\text{c}}-R_{\text{t}}\right)}^{2}{-\left(x-x_{0}\right)}^{2}}$ with *x*_0_ as *x*-coordinate of center of the circular arc/vesicle) to a line profile through the maximum of the particle and subtracting the tip size (Figure 4A). Hereby only data above half the maximum vesicle height is fitted, to take into account that only the upward-facing surface of particles is imaged in AFM. A simple alternative to extract the *R*_c_ using fewer data points is by extracting it directly from the following derivation:

(1) $$\begin{array}{l} R_{\text{c}}=\;\frac{FWHM^{2}+\;H^{2}}{4H}-R_{\text{t}} \end{array}$$

where *H* is the height, FWHM is the full width half maximum of the vesicle and *R*_t_ is the radius of the tip.

Finally, even when minimizing imaging forces, such forces will still deform soft samples such as vesicles. A deformation correction can be applied for accurate measurement of the size and shape of the vesicles on the surface. To this end, the deformation at the center of the vesicle can be measured by comparing the height obtained from the image with the height measured during nanoindentation (Calò et al., 2014) (Figures 4A,B). The imaging force also has a large impact on the measured radius of curvature (Figures 2C,D), and is in fact typically ~2.5 times larger than on the height (Vorselen et al., 2017). For a more accurate estimation vesicles can be imaged at increasing forces (Figure 2D).

From the radius of curvature and height of the spherical cap it is possible to approximate the original size of the vesicle *R*_0_ before surface binding.

(2) $$\begin{array}{l} R_{0}=\sqrt{R_{\text{c}}H_{\text{i}}-\frac{H_{\text{i}}^{2}}{4}} \end{array}$$

with *H*_i_ is the height obtained from the distance between the contact point and the substrate in the force indentation curve (see below and Figure 4B). Lipid bilayers can only strain by a few percent (Needham and Nunn, 1990), so the surface area—and not the volume—is expected to be conserved during spreading. This implies that upon binding to the surface the volume of the vesicle decreases, resulting in an outward osmotic pressure for natural vesicles or liposomes in salt solution. For severely flattened caps, the spherical cap shape predicts a sharp angle at the surface, which does not represent a physiological situation (Seifert and Lipowsky, 1990). For calculations of the surface area and volume, a rim with minimal radius of curvature 5–10 nm might be more realistic.

#### Analysis of Force Indentation Curves

**Figure 5:**
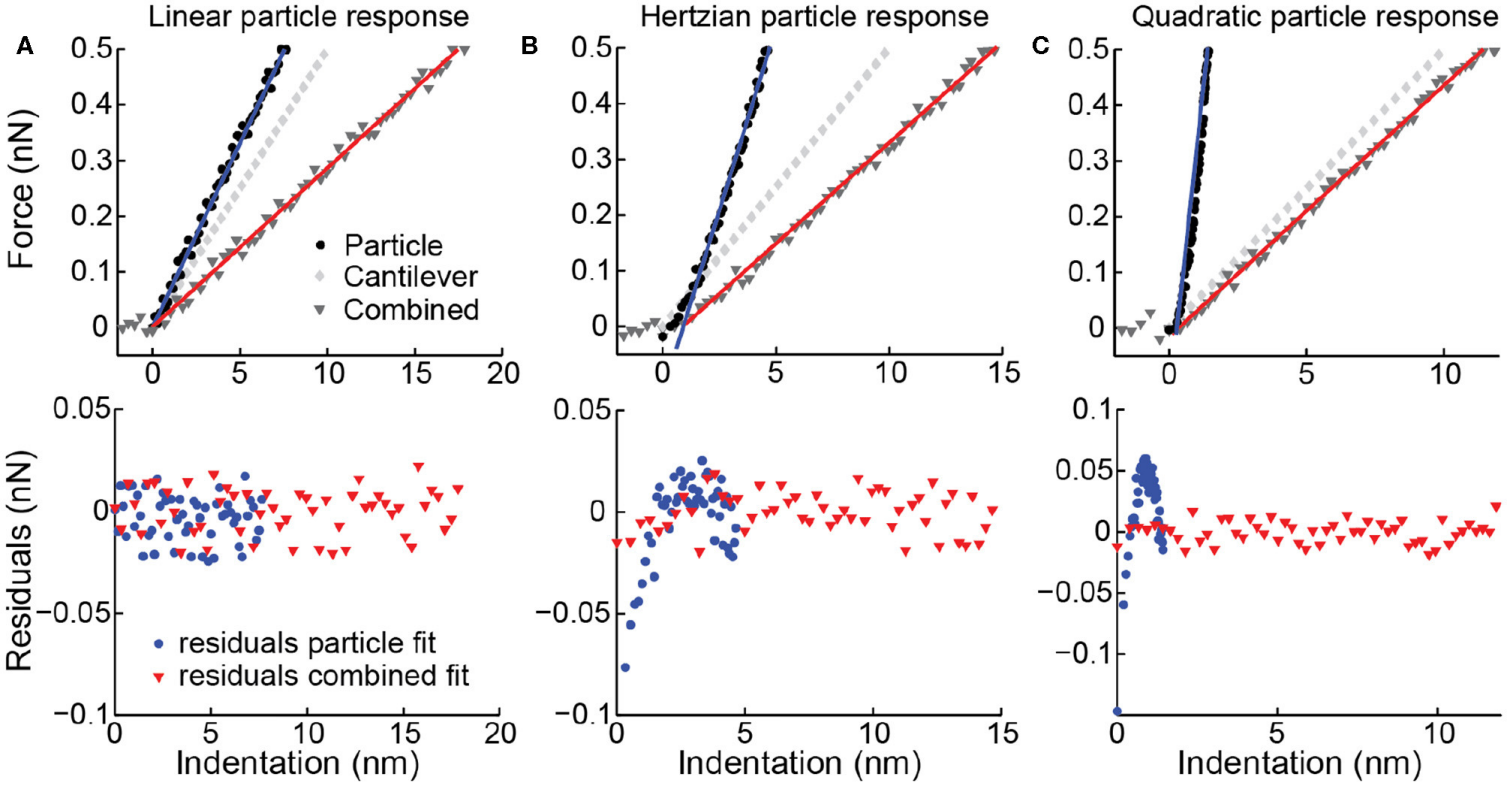
Simulated force distance curves (FZCs) and force indentation curves. Upper panels simulated deformation of a particle (corresponding to a force indentation curve), deformation of the cantilever and combined deformation (corresponding to an FZC) for three different particles. **(A)** Linear particle response with *k* = 0.067 N/m, **(B)** Hertzian response (0.05x^1.5^) and **(C)** quadratic response (0.25x^2^). Simulated spring constant of the cantilever is 0.05 N/m, and noise is simulated with std. of 10 pN. Linear fits to the indentation (blue) and combined data (red) shown as solid lines. Lower panels residuals belonging to the fits of the combined response (red triangles) and to the particle response (blue circles). **(B,C)** Residuals of FZC fits do not show a trend or deviation from a normal distribution (*p*-value of Kolmogorov-Smirnov (KS)-test 0.86 respectively 0.76), so the non-linearity of the particle response is not detected. Residuals of force indentation curve fits do show a clear trend and deviation from a normal distribution (*p*-value of KS-test 0.019 respectively <10^−10^), correctly indicating that the linear model is not applicable.

The force distance curves (FZCs) recorded during a nanoindentation experiment (approach and retract curve during one cycle) relate the AFM *Z*-piezo height to the recorded force and thus represents a combined response of the cantilever and the vesicle. Common practice in the data analysis of indentations of nanoparticles is fitting a linear function to this combined response (approach curve) to obtain the particle stiffness (Roos, 2011). This analysis should, of course, only be performed when the response of the particle is linear. A non-linear sample response may be masked by the cantilever response, especially in the case that the particle stiffness is higher than the cantilever stiffness (Figure 5). Therefore, it is advised to first subtract the cantilever response (see Equation 3), creating a force indentation curve (which relates the height of the AFM tip to the recorded force), and then fit the indentation response of the particle. In practice, this is done by making and fitting an FZC on a very stiff surface obtaining the cantilever stiffness (*k*_cantilever_). Then, for each data point in an FZC on a particle the force and hence cantilever deflection is known and can thus be subtracted to obtain a force indentation curve. By linearly fitting the approach curve for small indentations (e.g., 0.02–0.1 *R*_c_), the vesicle stiffness is obtained. The transformation from FZCs into force indentation curves is especially important for the analysis of vesicles, since both linear (Calò et al., 2014) and non-linear behavior (Liang et al., 2004b; Li et al., 2011) have been observed. We have previously shown that the vesicle indentation response can change from linear to non-linear in a vesicle-and tip-size dependent manner (Vorselen et al., 2016, 2017). For other nanoparticles, such as small viruses for which the thin shell model (shell thickness < < shell radius) does not apply, a linear response is not expected either. Hence, this approach for extracting force indentation curves can be beneficial in the study of many nanoparticles.

Transformation of force distance curve (FZC) in force indentation curve

1. Correct the baseline of the FZC for the offset in *x*- and *y*-direction.
2. Determine the slope of a recorded FZC on the substrate (“clean” functionalized poly-L-lysine -substrate).
3. Transform the FZCs acquired on a vesicle into a force indentation curve by transforming the *x*-coordinate with the following equation
  (3) $$\begin{array}{l} d=x_{\text{FZC},\text{ vesicle}}-\frac{F_{\text{vesicle}}}{k_{\text{cantilever}}} \end{array}$$

Extracting height, stiffness, and tether force of a vesicle from a force indentation curve

**Figure 6:**
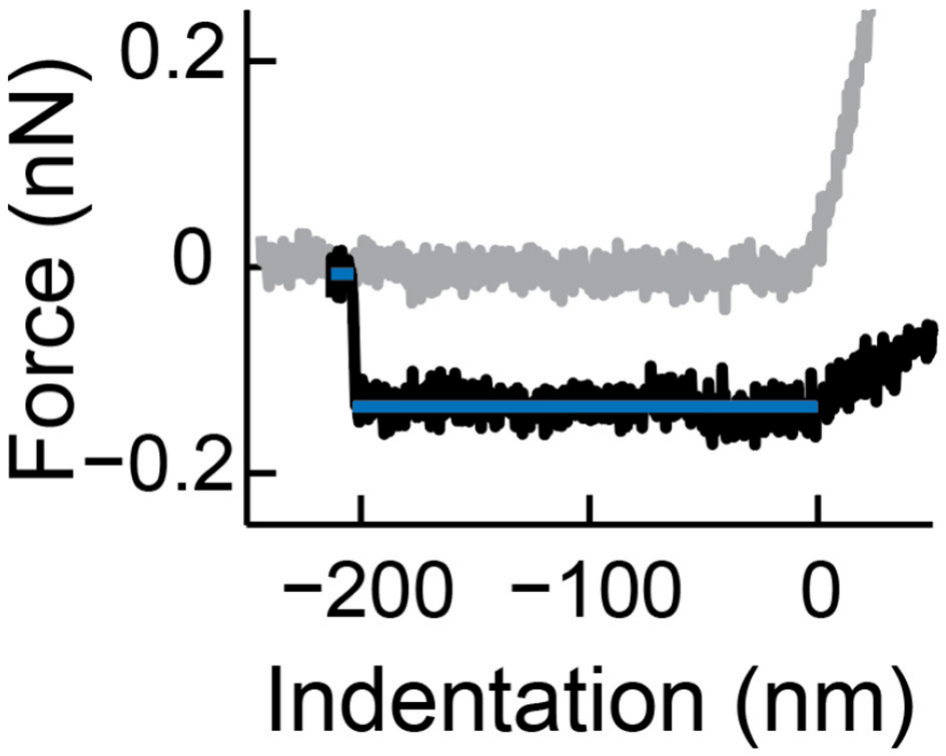
Determination of the tether force. Shown is a typical tether formed during a force indentation curve (approach in gray, retract in black). The difference of the forces of the two fitted regimes (blue lines) is the tether force. Reprinted from Vorselen et al. (2017) with permission from ACS.

1. First, the height of the vesicle can accurately be determined as the distance between the contact point (defined as 0 nm indentation) and the indentation when pushing on the substrate (infinite slope in the force indentation curve). Contact point can be found using, for instance, a change point algorithm.
2. Plot all force indentation curves [indentation in units of *R*_c_) acquired for one set of vesicles in one graph in order to determine the initial linear regime [e.g., 0.02–0.1 *R*_c_, see also (Vorselen et al., 2017)]. Within the chosen regime no superlinear behavior and no discontinuities should be observed for a fluid lipid vesicle.
3. By linearly fitting this regime in the individual force indentation curves (indentation in units of nm), the stiffness of each individual vesicle is obtained.
4. Identify all vesicles showing a tether in their retract curve. Importantly, a tether is only regarded as such if the length exceeds the contact point and shows a clear discontinuity related to detachment of the tether.
5. Obtain the tether force *F*_t_ of the last detachment of the tether (see Figure 6). Two lines with slope 0 can be fitted to the appropriate intervals in the retract curve and the tether force is obtained by the absolute value of the difference of the *y*-axis intercepts. Note: Typically, high tether forces (>250 pN) are excluded from further analysis (determination of bending modulus), as they could originate from tethers consisting of multiple bilayers.

Here, we use a model to analyze indentation curves based on Canham-Helfrich theory (Canham, 1970; Helfrich, 1973; Vorselen et al., 2017), which has been used extensively to describe the shape and deformation of giant unilamellar vesicles (GUVs, >1 μm; Bassereau et al., 2014; Dimova, 2014). This model predicts that the stiffness of vesicles with spherical cap shape under pure bending

(4) $$\begin{array}{l} k\;\approx \frac{27\kappa }{{R_{\text{c}}}^{2}}, \end{array}$$

where κ describes the resistance to bending of membranes, the bending modulus (Vorselen et al., 2017). Not surprisingly, the vesicle stiffness also depends on the membrane radius of curvature *R*_c_. In addition, a pressure difference over the membrane (ΔΠ) can contribute to the resistance to membrane bending and lead to increased vesicle stiffness. Due to surface adhesion, such pressurization is likely to occur in AFM-based measurements of vesicles, and, in fact, under physiological salt conditions the recorded mechanical response of adherent vesicles may be pressure dominated rather than bending dominated (Vorselen et al., 2017, 2018b). The vesicle stiffness obtained in AFM will thus be affected by the intrinsic resistance of the membrane to bending, which is quantified by κ, and vesicle pressurization ΔΠ, making independent estimations of these parameters difficult from the measured stiffness alone. To separate membrane bending and pressurization contributions to the vesicle stiffness, analysis of lipid tethers in the retraction curve of an indentation cycle is essential. The tether force *F*_t_ depends on both the bending modulus and the tension σ in the membrane, with $F_{t}=2\pi \sqrt{2\sigma \kappa }$, (Heinrich and Waugh, 1996; Cuvelier et al., 2005). The membrane tension, in turn, can be used to estimate the osmotic pressure difference over the membrane through the Young-LaPlace equation $\Delta \Pi =2\sigma R_{c}^{-1}$. Thus, tether force measurements establish a second independent relationship between the bending modulus, membrane pressure, and a measurable quantity (*F*_t_). Together, the stiffness and tether force can be used to estimate both the membrane bending modulus and vesicle pressurization.

In practice, the tether force *F*_t_ can be extracted as the last force plateau before the cantilever returns to the equilibrium position at 0 nN force (Figure 6). The bending modulus κ can then be obtained by fitting the data (stiffness, radius of curvature and tether force) to a previously derived numerical relationship between dimensionless pressure and stiffness relationship (Vorselen et al., 2017). In particular, κ is obtained by minimizing the sum of the squared log Euclidian distance between the experimental data and the theoretical curve. The error for κ can be obtained by bootstrapping. Finally, κ can then be used to obtain an estimate of the osmotic pressure over the membrane $\Delta \Pi =F_{t}^{2}{\left(4\pi^{2}R_{c}\kappa \right)}^{-1}$.

Determination of the bending modulus and estimation of the osmotic pressure difference over the membrane

1. Use the data for the theoretical relationship of normalized stiffness *vs*. normalized pressure from Vorselen et al. (2017).
2. Minimize the sum of the squared log Euclidian distance between the resulting curve and experimental values $\overset{n}{\underset{i=1}{\sum }}\underset{j}{\min}\left[\log{\left(\frac{F_{t_{i}}^{2}R_{c_{i}}^{2}4\pi^{-2}\kappa^{-2}}{x_{j}}\right)}^{2}\log{\left(\frac{K_{i}R_{c_{i}}^{2}\kappa^{-1}}{y_{j}}\right)}^{2}\right]$, where *x*_*j*_ and *y*_*j*_ are the values of the corresponding theoretical values for the normalized stiffness and pressure, with κ as single fit parameter. Please note that a sizable number of vesicles (~50) is necessary to obtain accurate estimates for κ.
3. The corresponding error of κ is obtained by bootstrapping (e.g., 500 repetitions), where in each bootstrap repetition an equal number of data points to the original sample size are randomly drawn from the sample and using this new sample κ is reevaluated.
4. Use κ obtained in step 1 to plot the experimental data together with the theoretical curve in a normalized stiffness *vs*. normalized pressure plot.
5. Use κ obtained in step 1 to estimate the osmotic pressure difference over the membrane $\Delta \Pi =F_{t}^{2}{\left(4\pi^{2}R_{c}\kappa \right)}^{-1}$.

#### Cantilever and Tip Selection

It is essential to make an appropriate choice for cantilever stiffness and tip size for the mechanical investigation of vesicles. First of all, for the best results in FZC-based AFM imaging, the cantilever resonance frequency in liquid should be at least ~5 times higher than the frequency at which the FZCs are recorded during imaging (Pfreundschuh et al., 2014). Currently, the speed of FZC-based imaging is often <1 kHz. Cantilever stiffness is also critical during indentation, and in general the stiffness of the sample should be comparable to the spring constant of the cantilever. A high cantilever spring constant may result in a low signal-to-noise ratio. However, if the cantilever spring constant is much softer than the sample, the observed combined response is mainly attributed to the cantilever (Figures 5B,C), and small percentage errors in determining the cantilever spring constant can cause major errors in the derived particle response. In our experience, cantilever spring constants in the range of 0.05–0.1 N/m are appropriate for working with fluid vesicles.

The theoretical response of vesicles to indentation is best described for an exerted point force (Vorselen et al., 2017). However, very sharp tips (~2 nm) can disrupt the integrity of vesicles; therefore, in our experience it is best to use tips with a radius 10–15 nm. These tips result in similar behavior during the initial part of the indentation of vesicles (*R*_c_ > 50 nm), while keeping vesicles intact. Larger tips also contribute to a larger broadening effect in the images, making size and shape estimates less accurate. Additionally, the tip size affects the indentation behavior of small vesicles: Indentations performed with larger tips result in an earlier onset of superlinear behavior compared to smaller ones (Vorselen et al., 2017; see section *Anticipated Results* for discussion of expected indentation behavior) and thus shortens the interval of the indentation curve that can be used for obtaining the spring constant of a vesicle.

An overall pyramidal tip shape with rounded apex works well in our experience for both vesicle imaging and indentation (e.g., qp-BioAC). Typically used cantilever and tip materials are silicon or silicon-nitride. However, other materials, such as quartz-like tips may work equally well. Special attention should be paid to the reflective coating of the cantilevers, as it should not interact with the sample nor the buffer used during experiments. Gold is a suitable coating working with vesicles.

### Anticipated Results

Depending on the sample used, vesicles might spread strongly or stay in a more spherical shape upon substrate adhesion. When image processing is automated, it is possible to obtain statistics on vesicle size and shape quickly. Natural vesicles often stay in a more spherical shape than liposomes (Vorselen et al., 2018b). Indentations often reveal complex and varied behavior. To characterize the initial behavior, individual force indentation curves can be fitted using an exponential function *bx*^α^, where *b* is a positive prefactor and α is the positive exponent. Plotting on a log-log scale can help identify the exponent of the force indentation curve. Expected behavior for fluid membranes is a linear to slight superlinear initial response (exponent α ≈ 1.05), which is clearly distinct from a Hertzian response expected for solid materials (α ≈ 1.5). The force curve is expected to subsequently flatten at an indentation of ~0.37 *R*_c_, which persists until the two lipid bilayers are pressed together, marked by a steep rise in force. Subsequently two discontinuities follow, corresponding to the penetration of the two lipid bilayers (Figure 4B; Vorselen et al., 2017). Natural vesicles show a similar response (Vorselen et al., 2018b). Sometimes more discontinuities occur, presumably related to either vesicle rupture (Vorselen et al., 2018b) or penetration of additional lipid bilayers in the case of liposomes (Vorselen et al., 2018a). Strong superlinear behavior (α ≈ 1.5–2) is observed when the tip radius is larger (~*R*_t_ > 0.25 *R*_c_) than the vesicle radius (Vorselen et al., 2017). For larger natural vesicles (>400 nm) or liposomes with internal structure, a contribution from the lumen of the vesicle and possibly Hertz-like behavior may be expected.

Often, after multiple wall-to-wall indentations of the vesicles, they still regain their initial shape, showing the remarkable ability of lipid bilayers to deform and recover (Li et al., 2011; Calò et al., 2014). For reliable statistics, ~50 vesicle indentations are needed for each condition. During a comparison of the vesicles, it is important not only to look at the stiffness, but also at the size and shape of the vesicles. Flattening of vesicles and resulting pressurization leads to increased stiffness. Preferably, vesicles should therefore be compared with vesicles of similar size and shape. Determining the bending rigidity of the vesicles, which is an intrinsic membrane property, overcomes the latter restriction and allows comparison between different populations of vesicles.

### Applications and Limitations of the Method

The procedure described in this paper is demonstrated for studying size, shape and mechanical properties of both liposomes (Vorselen et al., 2017, 2018a; Sorkin et al., 2020) and naturally excreted vesicles (Sorkin et al., 2018; Vorselen et al., 2018b). Recently, this approach was applied for liposomes with and without the proteins Syt1 (Synaptotagmin-1) and Doc2B (Double C2-like domain-containing protein beta) to study their effect on membrane mechanics (Sorkin et al., 2020). The procedure could easily be used to study the mechanics of similar-sized liposomes of varying compositions and natural vesicles from a wide range of sources. Such experiments can elucidate the role of lipid composition, membrane proteins and luminal proteins on vesicle adhesion and mechanics. Furthermore, buffer conditions can be varied, for example, for the study of the effect of pH on vesicle stability.

Vesicle adhesion is an essential aspect for vesicle nanoindentation experiments and can strongly affect vesicle mechanical measurements. In this approach, we use glass coated with poly-L-lysine in order to attach the vesicles electrostatically. The investigation of different types of vesicles, e.g., liposomes composed of zwitterionic phospholipids, may require the substrate coatings to be optimized and it may be necessary to use an entirely different coating strategy. Systematic investigation of the influence of poly-L-lysine density, lipid charge and ionic strength could reinforce the validity of the approach, and the model in particular, and could allow for a definition of optimal conditions (standardization) for the analysis of any given system. This protocol could in principle also work for larger vesicles (e.g., GUVs). However, a potential challenge for larger vesicles is the adhesion to the surface. Larger vesicles can yield smaller tension and may rupture when adhering to a surface. Furthermore, indentations must be performed slowly, such that water can diffuse through the membrane on the timescale of the indentation process.

Importantly, this approach is most suited for the study of vesicles with a membrane in the fluid state (presumably both disordered and ordered). For gel phase membranes, or those with multiple coexisting phases, a different data analysis strategy is likely more appropriate. As temperature can also critically affect the state of lipid bilayers and hence the mechanical properties of vesicles, assessing vesicle mechanics at various temperatures would be highly interesting. Moreover, experimental examination of vesicles at 37°C would likely better reflect their mechanics under physiological conditions. Although the experiments described here were all performed at room temperature, many AFM systems can be combined with temperature control, and the experimental procedure at 37°C would be similar to the one described here.

Another limitation of this procedure is that spreading and the resulting pressurization causes a strong increase in stiffness of the vesicle. The pressure can be approximated, but the resulting fit to obtain the bending modulus is only precise for a sufficiently large population of vesicles (Vorselen et al., 2017). This makes it currently only possible to use this procedure for accurate determination of the bending modulus of vesicles, when a sufficient number of vesicles is analyzed. Vesicle-to-vesicle variation in bending modulus within a vesicle population will hence be hard to detect.

## Discussion

Here, we presented a procedure to study size, shape and mechanical properties (stiffness, osmotic pressure and bending modulus) of single small vesicles under physiological salt conditions by AFM imaging, nanoindentation and subsequent analysis. While other methods for quantification of vesicle mechanics have been around longer and are therefore more established, this includes for instance fluctuation microscopy and micropipette aspiration, these methods all require imaging or manipulation at the micron scale (Bassereau et al., 2014; Dimova, 2014), and are hence inappropriate for the study of small (<200 nm) vesicles. This AFM-based approach is unique in that it can be used for the measurement of nanoscale vesicles, which are particularly abundant in biology. Other AFM-based approaches, such as AFM-based indentation of solid supported bilayers (Garcia-Manyes and Sanz, 2010), provide valuable membrane mechanical parameters and can uniquely report on local variation within a membrane. However, the obtained mechanical parameters are hard to relate to the membrane bending modulus (Loi et al., 2002; Garcia-Manyes and Sanz, 2010), which is often of particular interest as it describes the response to physiologically important membrane deformations. Moreover, such methods require vesicle rupture, and hence negate any potential contributions from luminal structures in biological vesicles.

Among other AFM-based approaches for vesicle indentations, the procedure described here is distinct in that it is based on Canham-Helfrich theory (Canham, 1970; Helfrich, 1973), and hence accounts for important well-established membrane properties, such as membrane fluidity. Other models have been previously used for interpretation of vesicle indentations, including the Hertz model (Laney et al., 1997; Liang et al., 2004a,b; Benne et al., 2020) and elastic thin shell models (Li et al., 2011; Calò et al., 2014; Takechi-Haraya et al., 2016). The Hertz model leads to obvious underestimation of elastic moduli, since it assumes a solid ball and not a thin membrane surrounding an aqueous lumen. Thin shell models ignore the bilayer fluidity, a characteristic membrane attribute, and therefore do not accurately describe the physical response of the vesicle to indentation. In particular, a fluid membrane has a negligible shear modulus and hence a similarly negligible Young's modulus. Moreover, both these models do not account for the pressurization upon substrate binding, which can lead to an overestimation of the bending modulus. Importantly, neglecting pressurization could even lead to misinterpretation in mechanical comparisons between vesicle types, especially if these vesicles spread differently.

**Figure 7:**
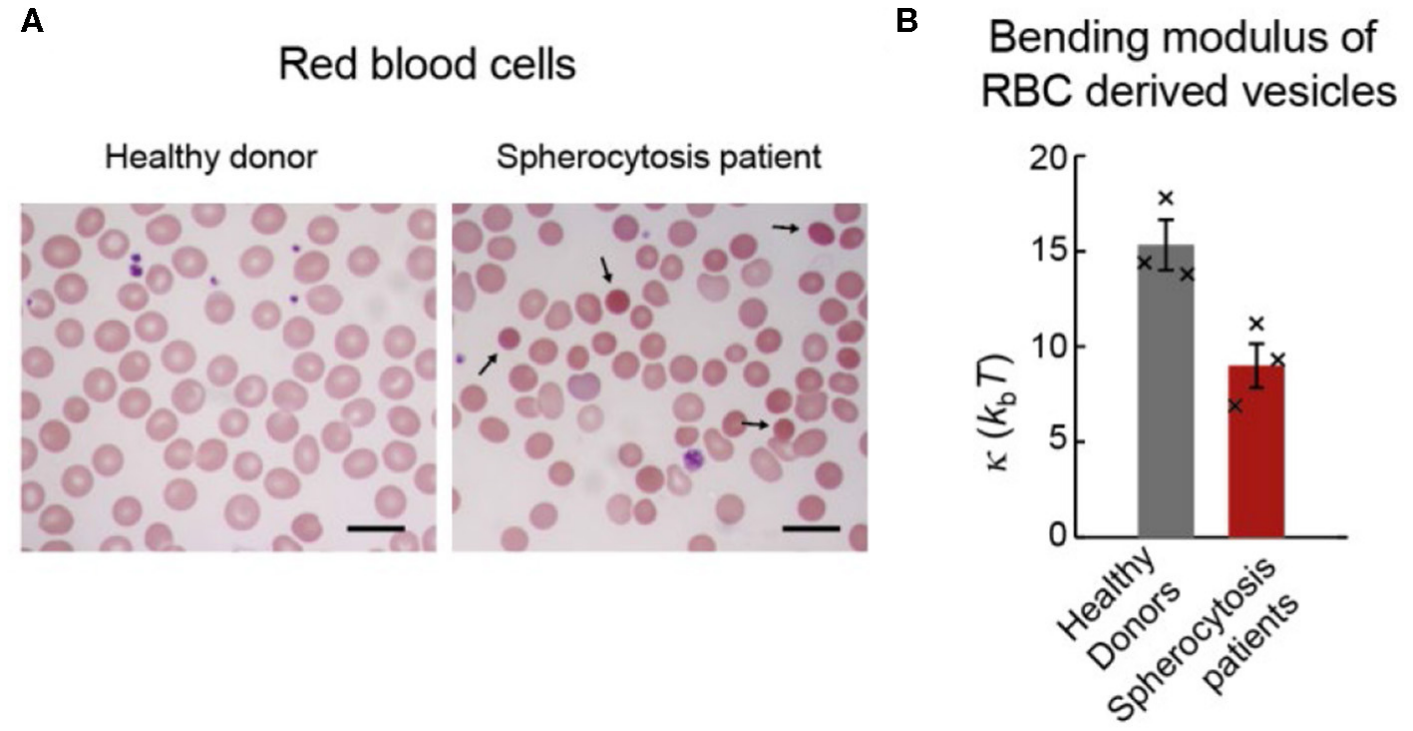
RBCs and EVs from healthy donors and spherocytosis patients. **(A)** Blood smears of RBCs stained with a May-Grnwald Giemsa stain. Left panel shows RBCs from healthy donors. Right panel shows RBCs from a hereditary spherocytosis patient; black arrows show typical spherocytes. Scale bar length is 10 μm. **(B)** Bending moduli of EVs from the three donors and the three patients. Histogram bars indicate means, and error bars indicate standard errors of the 3 sample means in each condition. Black crosses indicate bending moduli estimates for individual donor and patient samples. A two-sided *t*-test revealed statistically significant (*p* = 0.02) difference between the donor and patient groups. Reprinted from Vorselen et al. (2018b) with permission.

The procedure described in this paper was applied to naturally excreted vesicles (extracellular vesicles; EVs) secreted from red blood cells (RBCs) in healthy and pathophysiological states (Figure 7A; Sorkin et al., 2018; Vorselen et al., 2018b). It was found that RBC EVs from healthy donors had similar bending modulus (~ 15 *k*_b_*T*) as the RBC membrane, which is cholesterol-rich and potentially liquid-ordered, and as synthetic liposomes with similar lipid composition but without proteins (Vorselen et al., 2018b). Of note, unlike for the membrane of RBCs themselves (Rodríguez-García et al., 2015), there is no evidence that the membrane of RBC excreted vesicles is affected by metabolically regulated active cytoskeletal forces. Determining the bending modulus of RBC EVs from hereditary spherocytosis patients revealed a ~40% lower bending modulus compared to EVs from healthy donors (~8 *k*_b_*T*; Figure 7B). While the analysis of the lipid composition did not provide clear clues to explain this difference, the protein content of donor and patient EVs was significantly different and could potentially explain the different bending moduli. Due to the reduced linkage of the membrane with the underlying cytoskeleton, and thus reduced membrane organization, local accumulation of specific membrane proteins was hypothesized. The latter could, in turn, lower the bending modulus and the energy barrier for vesicle formation. This is in line with the reported increased vesiculation in patients with hereditary spherocytosis.

In another study the mechanical characteristics of EVs isolated from malaria-infected and non-infected RBCs was studied (Sorkin et al., 2018). No significant difference in their bending moduli was observed. Additionally, there was no obvious difference between the bending moduli obtained from RBC EVs and EVs produced by a certain type of tumor cell line (HT1080), and samples obtained from different donors yielded comparable results. On the other hand, the temperature conditions during incubation as well as different incubation times of the RBCs affected the bending modulus and the EV size. The fresher the sample, the softer the EVs. Taken together, the different bending moduli correlated with the total protein fraction estimated for the EV membrane, and this information was used to discuss potential ATP-level dependent vesiculation pathways taking place at different temperatures. Both these studies point to a role for membrane proteins in shaping the mechanics of small EVs. Although particular species or classes of membrane proteins (e.g., transmembrane proteins) could result in specific effects on membrane mechanics, such differences have not yet been identified for small vesicles. The lack of apparent contribution from the vesicle lumen to the mechanical response, as well as the relatively small amount of observed cytoskeletal proteins, at least in RBC EVs (Vorselen et al., 2018b), suggest no strong contribution of vesicle luminal proteins.

It has been shown that the results obtained with our method were independent of the isolation protocol of the EVs (Sorkin et al., 2018). The influence of the isolation method on measurements in general is vigorously debated in the vesicle community (Witwer et al., 2013). Next to applying the described procedure to vesicles from natural sources, it has also been used for the mechanical characterization of artificially produced liposomes (Vorselen et al., 2018a; Sorkin et al., 2020). It can for instance distinguish between unilamellar and multilamellar vesicles (Vorselen et al., 2018a). This approach was demonstrated for multilamellarity up to 5 bilayers, which was verified by cryo-electron microscopy measurements. With increasing lamellarity, the particles become stiffer and more spherical in shape. In particular, a linear correlation between the degree of lamellarity of the liposomes and the stiffness was found (~2.7 × 10^−3^ Nm^−1^, or 20% of the unilamellar vesicle stiffness, per added bilayer). Recently, the method was applied to liposomes with and without added integral membrane proteins to study the protein's influence on the mechanical properties (Sorkin et al., 2020). In this study, calcium-sensor proteins, Syt1 and Doc2b, and their interactions with the membrane were studied by optical tweezers and AFM-based nanoindentation. The AFM studies revealed that both proteins, Syt1 and Doc2b, reduce the bending modulus upon addition to the liposomes. This indicates that the insertion of the protein's C_2_AB domains into the liposome membrane effectively lowers the energy barrier for calcium-induced membrane fusion.

The wide-spread applicability of the method presented in this paper underlines its strength, however, for the moment it lacks high-throughput characteristics. The method is rather elaborate, and a large number of vesicles is needed for good statistics. The vesicles used for the determination of bending modulus need to fulfill certain criteria: they have to show tether formation in the retract curve, a good glass curve prior indentation of the vesicle and higher height extracted in the force indentation curve than in the image cross section. Since not all vesicles fulfill these requirements, a considerable number of particles need to be indented.

In order to increase throughput of the vesicle nanoindentation experiments, high-speed AFM (Ando, 2017; Marchesi et al., 2018; Maity et al., 2019) could potentially be used, although this has not yet been reported. Attempts to increase throughput have been reported in imaging studies. In such approaches the mechanical properties of small vesicles are studied solely by analysis of the AFM images, without resorting to nanoindentation. These methods are based on extraction of the contact angle between the vesicle and the substrate upon particle adhesion (Ridolfi et al., 2019), or on analyzing the increased deformation of vesicles for increasing imaging force (Alqabandi et al., 2019). These methods are relatively fast and can be used to provide rough information on the mechanical properties of the particles of interest. However, if values for bending moduli are needed, or when differences between particles are small, these methods are less appropriate and one may need the nanoindentation approach as described here.

As small (<200 nm) vesicles are abundantly present in cell biology and artificial vesicles of this size are used as drug delivery vehicles, studies on vesicle spreading and mechanics are important to understand how vesicles respond to mechanical stresses. This contributes to a fundamental understanding of their interaction with cells. Here, we presented the procedures for AFM-based characterization of the mechanics of small vesicles. This methodology, which has already been validated in several studies, marks important improvements compared to previous approaches. The improvements are both in terms of control during the experiments as well as in data analysis. It allows for reliable and reproducible quantification of the material properties (size, shape, stiffness, bending modulus) of vesicles obtained from different sources and for comparison between different studies. Furthermore, these procedures can be used to understand how the luminal and membrane composition influences mechanical and adhesive properties of both natural and artificial vesicles in various conditions. Herewith, an important step toward the standardization of the mechanical characterization of small vesicles has been obtained, and aspects of the general methodology can also be applied to various other nanoparticles.

## Data Availability Statement

The original contributions presented in the study are included in the article/supplementary material, further inquiries can be directed to the corresponding author/s.

## Author Contributions

DV, MP, WR, and GW wrote and reviewed the manuscript. All authors contributed to the article and approved the submitted version.

## Conflict of Interest

The authors declare that the research was conducted in the absence of any commercial or financial relationships that could be construed as a potential conflict of interest.

## Abbreviations

AFM: Atomic Force Microscopy/Microscope
EVs: Extracellular vesicles
FZC: Force distance curve
FWHM: Full width at half maximum
GUVs: Giant unilamellar vesicles
HCl: Hydrochloric acid
RBC: Red blood cell.
